# Impact of Recycled Aggregate on the Mechanical and Environmental Properties of Concrete: A Review

**DOI:** 10.3390/ma15051818

**Published:** 2022-02-28

**Authors:** Andrea Piccinali, Alessandra Diotti, Giovanni Plizzari, Sabrina Sorlini

**Affiliations:** Department of Civil, Environmental, Architectural Engineering and Mathematics, University of Brescia, 25123 Brescia, Italy; a.diotti@unibs.it (A.D.); giovanni.plizzari@unibs.it (G.P.); sabrina.sorlini@unibs.it (S.S.)

**Keywords:** construction and demolition waste, recycled aggregates, compressive strength, durability, w/c ratio, workability, water absorption, environmental properties

## Abstract

This review aims to present and discuss the mechanical and environmental properties of two different type of recycled aggregates obtain from construction and demolition waste (CDW): (1) Recycled Concrete Aggregates (RCA) and (2) Mixed Recycled Aggregates (MRA). In addition, the properties of the concrete in the fresh (workability, water/cement ratio) and hardened state (mechanical and durability properties), as well as the environmental impact of the concrete produced with the two types of recycled aggregates, are presented and discussed. Due to the heterogeneous composition of recycled aggregates, the concrete properties can be significantly variable. The systematic review concerns scientific papers published from 2010 to 2020 and it shows the importance of the selection process in order to obtain high quality CDW as well as of the type of recycled aggregates on concrete properties. In particular, recycled concrete aggregates show a better quality and homogeneity than mixed recycled aggregates that make them more suitable for concrete. This work presents an overview on the influence of recycled aggregate quality on the physical, mechanical and environmental properties of concrete.

## 1. Introduction

The construction sector in Europe uses about 50% of the total available raw materials, thus consuming huge quantities of natural resources and soil from which this material is extracted [1]. At the same time, according to Eurostat [2], the construction industry is also the largest waste producer as construction and demolition wastes represent about 35% of the total wastes produced.

This situation is also reflected at Italian level, where the construction sector in 2019 produced about 69 million tons of construction and demolition wastes (CDW) [3], representing about 50% of the total amount of special wastes produced at national level.

During the last few years, many European Countries, no longer having the possibility to dispose of this type of waste in landfills, and having a lack of extractable quarry material, have encouraged the development of recovery processes to transform these wastes into secondary raw materials that can be reused in the construction sector [4].

The practice of reuse has also been promoted in Europe by the Directive 2008/98/EC, which required Member States to reach a minimum percentage of recovery of CDW of 70% by 2020.

Many European countries have exceeded this minimum target, even reaching levels of 100% as in the case of The Netherlands, or 98% as the United Kingdom [5]. Italy for several years has exceeded this rate, with a constant increase over the years, until reaching 78% in 2019 [3]. Unfortunately, despite the high recovery rate, the reuse of these materials in the construction sector for structural elements is limited [6]. In fact, only 7% of the recycled aggregates (RA) produced is used as an alternative to natural aggregates in concrete production, while the remaining 93% is used as a road substrate or for filling operations, depending on their characteristics like the origin of the aggregates, and the mechanical and environmental properties [7,8].

The Italian legislation is trying to promote the use of recycled materials in the production of concrete by means of the Legislative Decree 11 October 2017 which has made mandatory the use of at least 5% of recycled material in concrete structures for public procurements. Furthermore, the use of RA as total or partial substitute of natural aggregate is also allowed by the Italian structural code, which limits the percentage of natural aggregates replacement, depending on the concrete grade and its possible structural applications [9].

The final use of RAs is related to their specific characteristics. According to Silva et al. [10], two main types of RAs can be recovered from CDW: (1) Mixed Recycled Aggregates (MRAs), and (2) Recycled Concrete Aggregates (RCA). The first one, which is produced in higher quantity and is strongly heterogeneous, can hardly be used in structural concrete [11,12,13,14]. Conversely, RCAs are expected to be used to produce structural concrete as these have a minimum content of recycled concrete of at least 90%, due to the lower heterogeneity and better mechanical characteristics [15,16]. However, since the RCAs consist of original aggregate and mortar, they can be considered as similar materials, but their properties are generally different since they depend on the properties of the original concrete: It should be underlined that concrete made with RCAs not only decreases the mechanical properties and durability performance but also reduces the density and workability at the fresh state [17].

The fears of end users, nowadays, make it difficult to apply these materials in new construction, therefore, increasing their knowledge, and demonstrating how the required technical and environmental criteria are respected, how it can be used and what are the results obtained is an important objective.

Assuming that characteristics are worse, in this research, we wish to analyze the results obtained in the numerous studies proposed in the last 10 years, showing how concrete produced with these types of aggregates still has good applicability and how the quality of these aggregates allows higher value uses compared to fillings and road foundations. Many literature studies on this topic are presented, but given the high heterogeneity of the RAs, the results on the mechanical properties of the concrete are very variable.

This article aims to perform a statistical analysis of a large amount of data, examining the variation of the data and find correlations between RA type and concrete properties. Furthermore, it aims to analyze how the quality of the RA can affect the mechanical end environmental properties of concrete and the importance of applying a suitable preliminary treatment that allows one to obtain selected RCAs will be discussed.

## 2. Materials and Methods

### 2.1. Study Selection, Elegibility and Search Strategy

The analysis presented herein was developed on the basis of the Preferred Reporting Items for Systematic Reviews and Meta-Analyses (PRISMA) statement [18]. The procedure consists of a 27-item checklist and a flow diagram [18] that helps to develop a structured review.

Studies were eligible if they met specified criteria. In particular, the paper selection was carried out in relation to the selected keywords presented in Table 1. Other types of aggregates or other recycled materials, like steel slag or fly ashes, were excluded in order to focus the review exclusively on CDW.

To be eligible for inclusion, studies must be published in scientific papers or review papers in order to analyze only reliable and verified results, thereby conference papers, book chapters, conference review books and editorials were excluded. Moreover, the selected papers must have been published in English between 2010 and 2020, which corresponds to 66% of the total studies found when placing no limitations.

The search for eligible studies was conducted using Scopus as a search engine with the combination of keywords listed in Table 1. After an initial screening of study titles and abstracts, each full paper was examined for eligibility. Subsequently, data were extracted from the eligible studies and adequately processed and analyzed.

### 2.2. Data Analysis

Different methodologies for graphical representation were applied for the analysis of the results of this research. All the results were analyzed and processed using the IBM SPSS software (version 19, IBM SPSS, USA). For all the results represented, an overview of the amount of data used in the study was initially given. This graphical representation has been carried out by using histograms representing the frequency of the analyzed values. This allows a first analysis of the data by evaluating which are the most representative cases.

**Figure 1:**
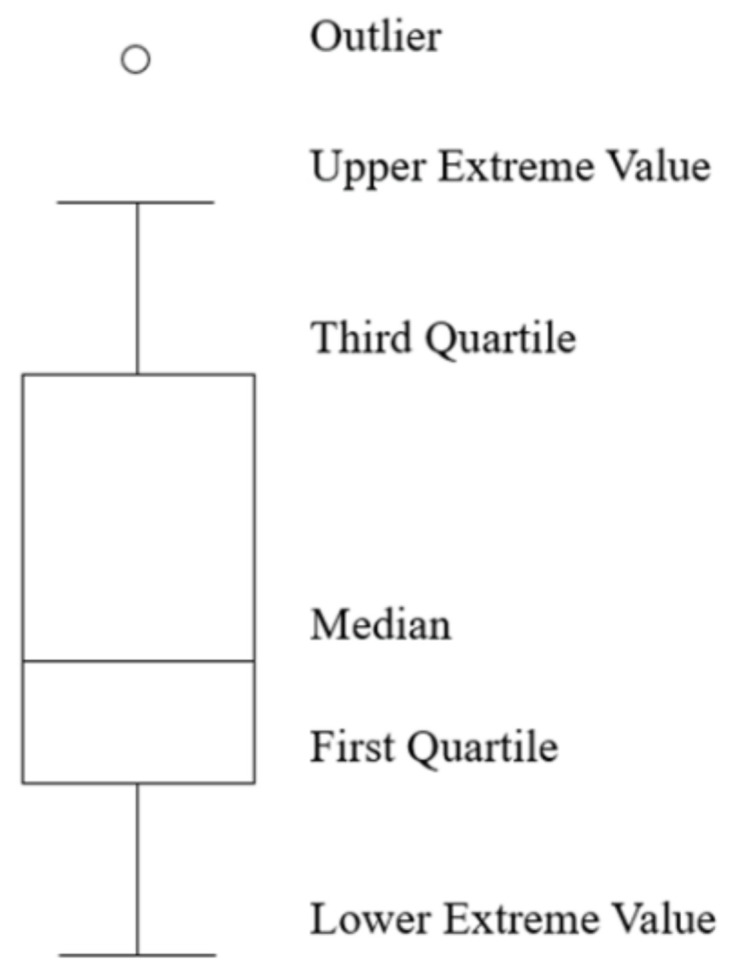
Box plot scheme.

The second methodology was based on a box plot according to the representation of Figure 1. The box is used to define the values of the first and third quartiles, and the interquartile range (IQR), which represents 50% of the data population. The horizontal line inside the box, called the median, represents the second quartile. Two vertical lines, called whiskers, are extended to the extremes of the distribution, and are the minimum and maximum values of the data population. The dots represent the outliers of the population [11].

Lastly, the third and last method used is the scatterplot, which is used for the graphical representation of the mechanical and physical properties analyzed in the research. Scatterplots are used to determine the intensity of a relationship between two numerical variables. The x-axis represents the independent variable, and the y-axis represents the dependent variable.

### 2.3. Study Selection

Initially a total of 5122 studies were identified. After the first screening and deletion of studies falling into the categories of book chapter, conference reviews and articles and keeping only published papers and reviews, 3720 studies remained.

After applying the second eligibility criterion, which provides for the sole inclusion of studies published from 2010 to 2020, the number of eligible studies was reduced from 3720 to 3406. Finally, after excluding non-English published studies, the final number of eligible studies was 3085. After removing duplicates, 1682 scientific articles remained. Of these, about 1000 studies were discarded after reviewing the titles and abstracts as these papers did not match the target of the study.

Regarding the 1000 discarded papers, they either did not meet the requirements or did not refer to the keywords used to find them. In fact, most of the studies were not related to concrete produced with Ras at all, but rather to other types of aggregates obtained from other recycled materials. Other studies were discarded because the main topic was a statistical analysis and not actual tests on concrete cubes or cylinders. The last inclusion criterion led to all the papers having as topic road and rail construction being discarded.

**Figure 2:**
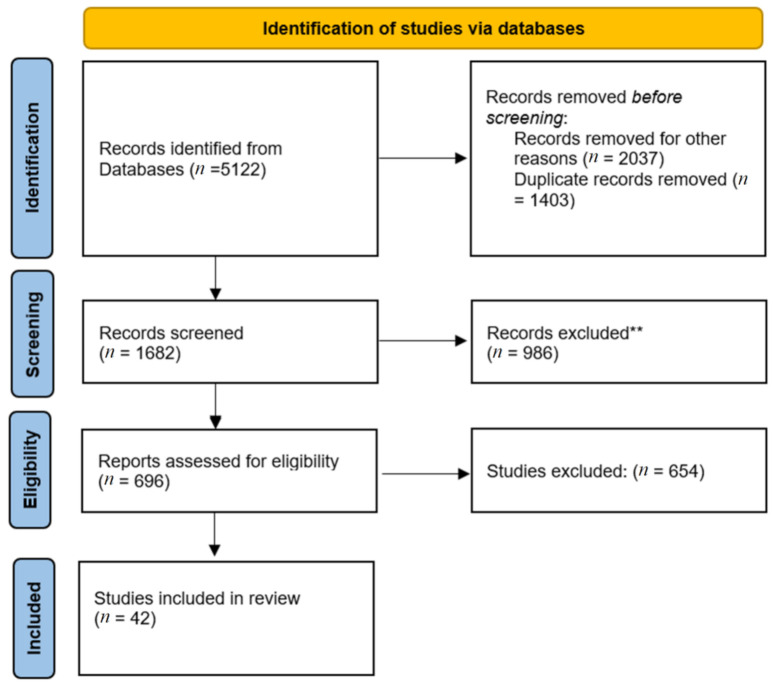
PRISMA flow diagram summarizing the study’s article selection.

The full text of the remaining 680 publications was examined in detail. A total of 640 studies did not meet the previously described inclusion criteria, while 42 studies did meet the inclusion criteria and were analyzed in this review. The corresponding PRISMA flow chart is presented in Figure 2.

## 3. Results

### 3.1. Water Absorption of Reycled Aggregates

**Figure 3:**
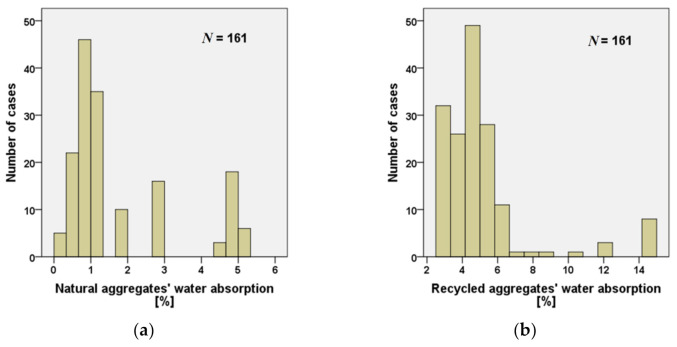
Number of water absorption data analyzed for (**a**) natural aggregates and (**b**) RAs [20,24,25,26,27,28,29,30,31,32,33,34,35,36,37,38].

Water absorption of RAs is a fundamental characteristic that must be considered in the mix-design of concrete-containing RAs. Several characteristics directly depend on this factor. In particular, compared to natural aggregates, the high water absorption of RAs strongly affects the concrete workability and everything that is directly related to it, such as the water/cement ratio (w/c ratio), the compressive strength and durability properties of concrete [19,20,21,22,23]. The graphs in Figure 3 show the amount of data analyzed regarding water absorption of RAs and natural aggregates, respectively.

In both cases, 161 specimens were analyzed. As shown in Table 2, the experimental data demonstrates a clear difference in water absorption between the two types of aggregates. In fact, the latter shows the average water absorption value for natural aggregates is markedly lower than the one of RAs, and the maximum and minimum absorption values also follow the same trend. Furthermore, from the histograms in Figure 3a it can be observed that about 48% of natural aggregates have a water absorption lower than 1%, while for the remaining 52%, 25% have an absorption value lower than 2%. On the contrary, 68% of RA have a water absorption lower than 5% and about 50% of the 161 analyzed values are in the range between 4% and 5% (Figure 3b).

This confirms the significant difference in water absorption between the two types of aggregates. Generally, this increase in water absorption is due to the adherent mortar which is responsible for the porous structure of the RAs [22,31,37,39,40,41,42,43]. Another reason is the presence of microcracks on the surface of the RAs caused by the crushing process [44,45].

In order to decrease the water absorption, Pedro et al. [46] stated that increasing the crushing process could reduce the amount of attached mortar. This consideration was also confirmed by Nagataki et al. [44] who stated that, by increasing the crushing process of RAs up to three times, the quality of the recycled aggregate can be improved.

**Figure 4:**
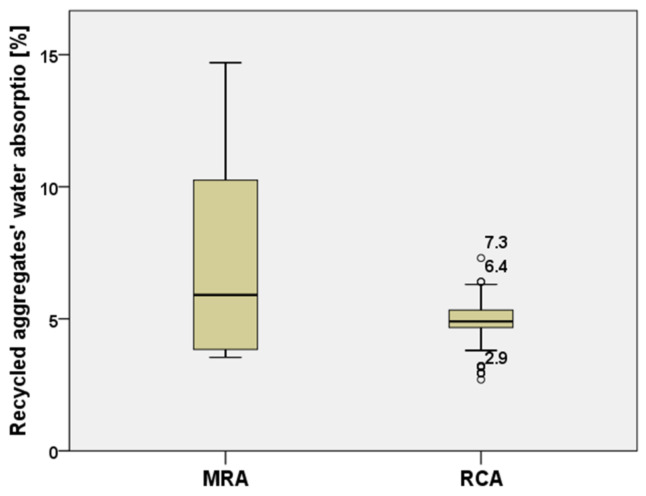
Comparison of water absorption values between MRAs and RCAs [20,24,25,26,27,28,29,30,31,32,33,34,35,36,37,38].

Moreover, the water absorption data (161 specimens analyzed for both types of RA) were compared between MRAs and RCAs, as shown in Figure 4. It can be observed that, MRAs have a high dispersion, as data are distributed between 4% to 15%, and 50% of them vary between about 4% and 8%. On the contrary, RCAs show a clear lower dispersion having 50% of the data concentrated around the median value of 5%. This result agrees with several studies showing that RCAs, have less water absorption than MRAs due to a lower presence of masonry or mortar [45,47,48]. Moreover, some values, classified as outliers, represented by circles, can have slightly higher and lower values with respect to the median value.

Ras’ water absorption depends on the aggregates’ size as well as the strength of the initial concrete. In fact, a lower aggregates size leads to an increase of porosity on the aggregate surfaces and, consequently, to an increase in the water absorption. The same occurs with the strength class of the original concrete; in fact, RCAs from high strength concrete have a less porous structure on the surface of the aggregates which leads to a decrease of water absorption [23,31,49,50,51]. Due to the high-water absorption of the RAs, additional water is necessary for concrete mixing in order to guarantee the required concrete workability [52].

### 3.2. Water/Cement Ratio of Concrete with Recycled Aggregates

**Figure 5:**
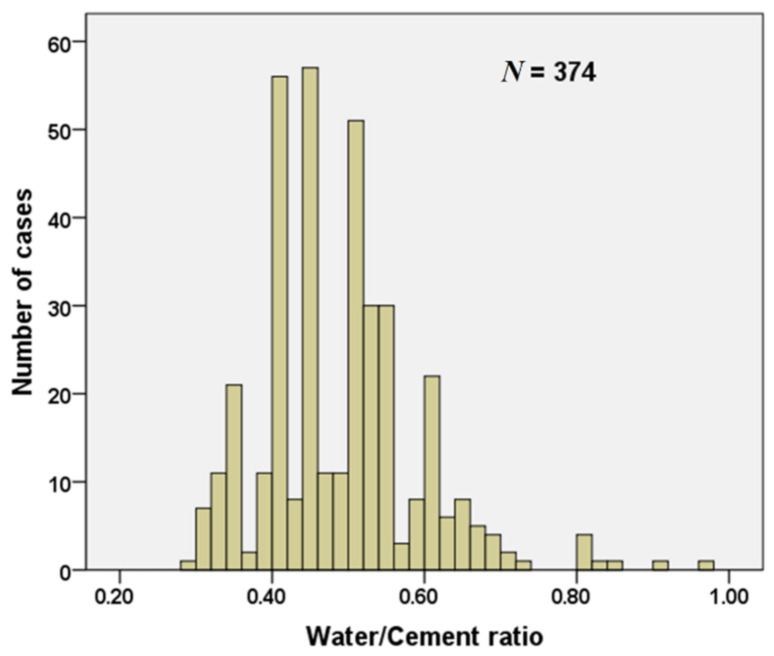
Number of data of water/cement ratio for concrete mixtures containing RAs [19,20,21,23,24,25,26,27,28,29,30,31,32,34,35,36,41,43,53,54,55,56,57,58,59,60,61,62,63,64,65,66,67,68].

The water/cement (w/c) ratio is an important parameter for the concrete mix design, and it is strictly related to the water absorption of RA. Figure 5 presents the specimens (total number of 374) with RA providing information on the w/c ratio.

It can be observed that the w/c values vary from a minimum of 0.29 up to a maximum of 0.97, and the most studied values are 0.40, 0.45 and 0.50, corresponding to the most representative values for construction applications.

**Figure 6:**
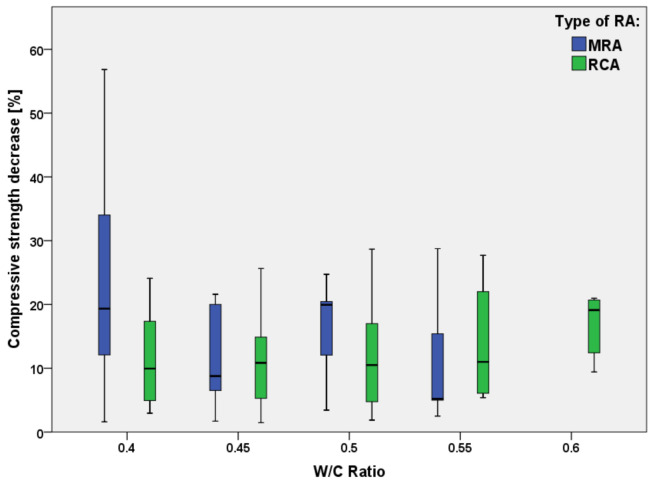
Decrease of compressive strength of concrete with RAs with respect to natural concrete with different water/cement ratios [19,20,21,23,24,25,27,29,31,34,41,55,56,57,58,59,60,61,67,68].

Figure 6 shows the decrease of compressive strength of the concrete mixtures produced with MRAs and RCAs, as compared to concrete produced with natural aggregates, for the w/c ratios most commonly used in construction (0.4–0.6).

It can be noticed that, for w/c ratios of 0.4, 0.45 and 0.5, the concrete produced with RCAs (green boxes) has a lower reduction of compressive strength than the corresponding concrete manufactured with MRAs.

For these three w/c values, the decrease of compressive strength for concrete made with RCAs is about 10% (average value), while for MRAs it varies from 10 to 20%. Data for a w/c ratio of 0.6 refer to RCAs only; it can be noticed that the average value of the compressive strength decrease is around 20%.

In summary, the relationship between the compressive strength and w/c shows high variability in the case of concrete with MRAs, probably due to their heterogeneous characteristics, especially for water adsorption.

The experimental scatter can be due also to the addition of superplasticizers used for concrete workability, which enhances the mechanical properties. For instance, Wagih et al. [69] stated that the use of superplasticizer could decrease the w/c ratio by 12% which, consequently, produces a lower compressive strength decrease.

### 3.3. Workability of Concrete with Recycled Aggregates

**Figure 7:**
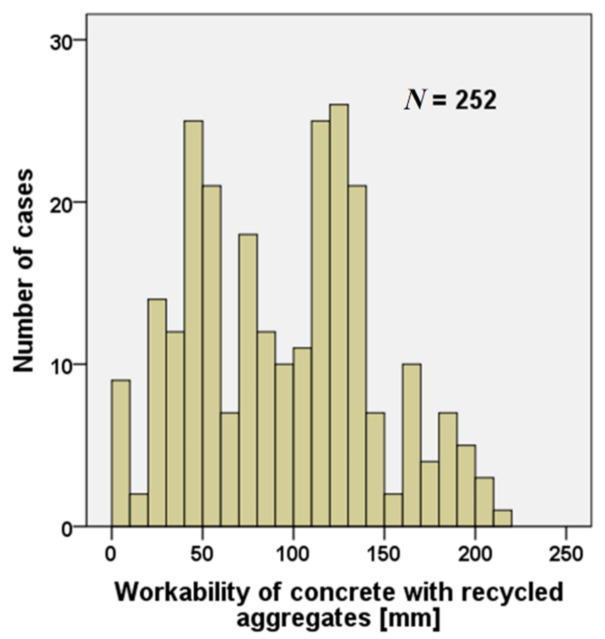
Number of cases of slump of concrete made with RAs analyzed [19,20,21,23,24,25,27,29,30,31,32,34,35,54,57,63,65,70].

An important property for fresh concrete, that has a strong influence on mechanical characteristics and durability, directly connected also to the water absorption and water/cement ratio, is the workability of concrete [20,28]. Figure 7 presents the values related to the workability of concrete containing RAs.

A total of 259 workability values according to UNI EN 206) [71] were analyzed. The studies evidenced two different types of approaches. In the first approach concrete mix designs were carried out in order to obtain a certain target workability [64,72], while in the second case, the mix design did not take it into account the workability; the latter was simply measured as a fresh concrete property.

The analysis of the literature shows that, among the several factors that affect the workability of concrete, water absorption is the most important one [31,55,62]. In fact, concrete workability is strictly related to the degree of substitution; high levels of substitution are related to low workability values [19,24].

Due to the high-water absorption of the RAs, RAs are often pre-saturated [25,29,31] in order to obtain the target workability without adding high water amount. However, Hentges et al. [73] stated that the use of pre saturated aggregates could increase the amount of water and the w/c ratio in cement mixture, thus decreasing its mechanical properties.

**Figure 8:**
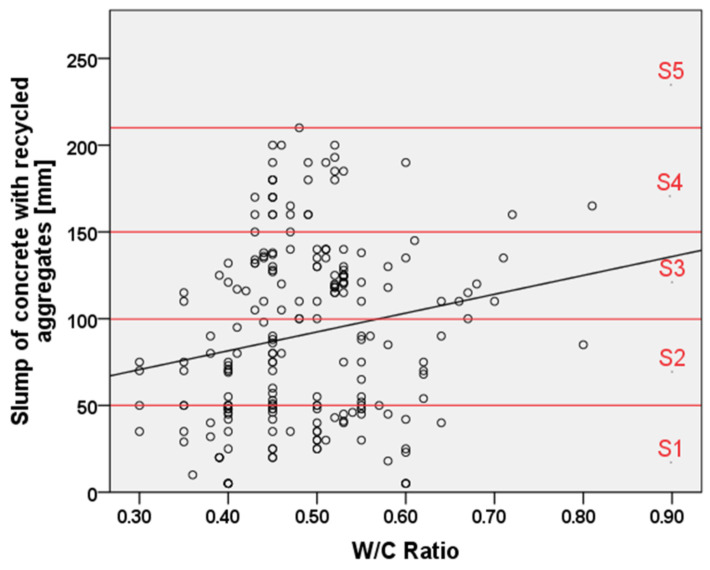
Relationship between water/cement ratio and the slump of concrete with RAs and workability classes expressed by the red horizontal lines [19,20,21,23,24,25,27,29,30,31,32,34,35,55,57,63,65,70].

Figure 8 shows the slump of concrete with RAs vs. the w/c ratio with evidenced the different consistency classes (from S1 to S5 as listed according to UNI EN 206) [71]. As expected, it can be noticed that a w/c ratio increase leads to a corresponding increase of concrete workability [20,22,31] as well as the experimental scatter and high workability can be obtained with typical w/c ratios of 0.45–0.55.

The easiest way to enhance concrete workability is the addition of allowed superplasticizers, even for large levels of substitution, achieving slump values close to those of the reference concrete [61,62].

As stated by Sri et al. [74] and Verian et al. [75], another possible solution to obtain the same level of workability could be the addition to the recycled concrete mixture of about 10–15% of additional water (with respect to the natural aggregates mixtures).

### 3.4. Compressive Strength of Concrete with Recycled Aggregates

Different factors can affect the mechanical compressive strength, including water absorption, the strength of the original concrete used to produce RAs, the type of RAs used in the mixture, the quality of these as well as the w/c ratio.

All the results refer to the compressive strength at 28 days, determined on cubic samples, despite the fact some tests were carried out on cylindrical samples (f_ck_) and transformed into cubic compressive strength (R_ck_), assuming the following conventional equation:

R_ck_ = f_ck_/0.83 (1)

**Figure 9:**
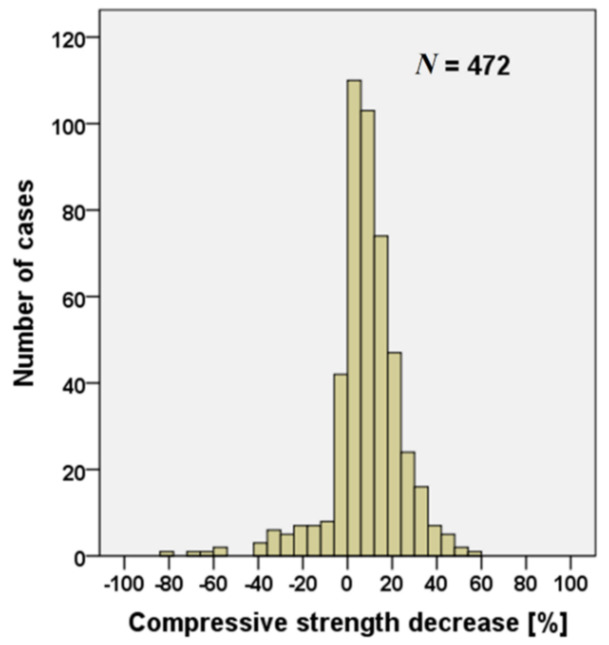
Number of data of compressive strength decrease for concrete mixtures containing RAs [19,20,21,22,23,24,25,26,27,28,29,30,31,32,33,34,35,36,37,38,41,42,52,53,54,55,56,57,58,59,60,61,62,63,64,65,66,67,68,70,72].

Figure 9 shows the decrease of compressive strength for concrete containing RAs compared to the same concrete mixtures including only natural aggregates (472 values are available). It can be noted that about 80% of the values show a decrease of the compressive strength of concrete with RAs. In specimens with a compressive strength increase, concrete workability was not reported in the following analysis.

**Figure 10:**
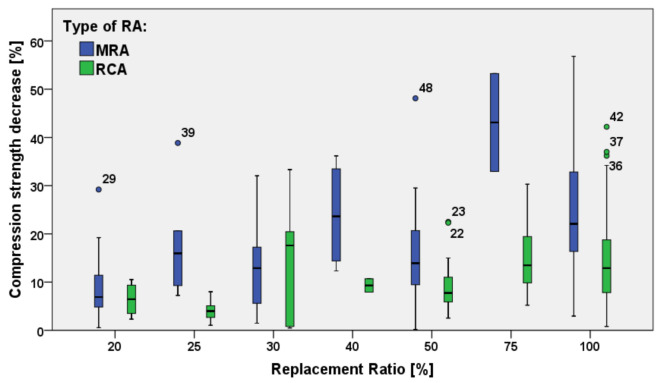
Comparison between MRAs and RCAs compressive strength decrease [19,20,21,22,23,24,25,26,27,28,29,30,31,32,33,34,35,36,37,38,41,42,52,53,54,55,56,57,58,59,60,61,62,63,64,65,66,67,68,70,72].

Considering the specimens with a decrease of compressive strength, 34% of them have a decrease lower than 10%, while a further 34% of the specimens have a decrease lower than 25%. Figure 10 shows the compressive strength decrease related to the type of RAs used for the concrete production.

It can be observed that concrete mixtures produced with RCAs have a lower decrease of compressive strength than concrete produced with MRAs. In addition, for all the replacement ratios analyzed (except for the case of 30% replacement), the median reduction of compressive strength of concrete with RCAs is lower than that of concrete with MRAs.

The high variability of values for MRAs concrete, with respect to RCAs concrete is noteworthy. This is due to a lower water absorption of RCAs as compared to MRAs. 

This statement is confirmed by Guo et al. [76] who stated that RCA concrete has less compressive strength decrease than concrete with MRA.

It should be also observed that, during mixing, RAs from CDW tend to crumble into finer particles, with higher water absorption, and make the gradation of the cement mixture finer, which, subsequently, provokes a further decrease of concrete strength [56,77].

As mentioned above, there are several factors that influence the decrease of compressive strength. As Thomas et al. [24] stated, the strength of concrete with RA depends on factors like the cement content, the original strength of the recycled aggregate and the interfacial transition zone between aggregates which is weaker due to attached mortar, when using RAs instead of natural aggregates.

Bidabadi et al. [30] and Rashid et al. [31] confirmed this statement, as in fact poor quality bonding between new cement paste and previous mortar tends to affect concretes’ compressive strength.

According to Eckert et al. [26], in order to decrease the attached mortar on the surface of RA, and to increase the quality of RAs an intensive crushing process could be applied.

**Figure 11:**
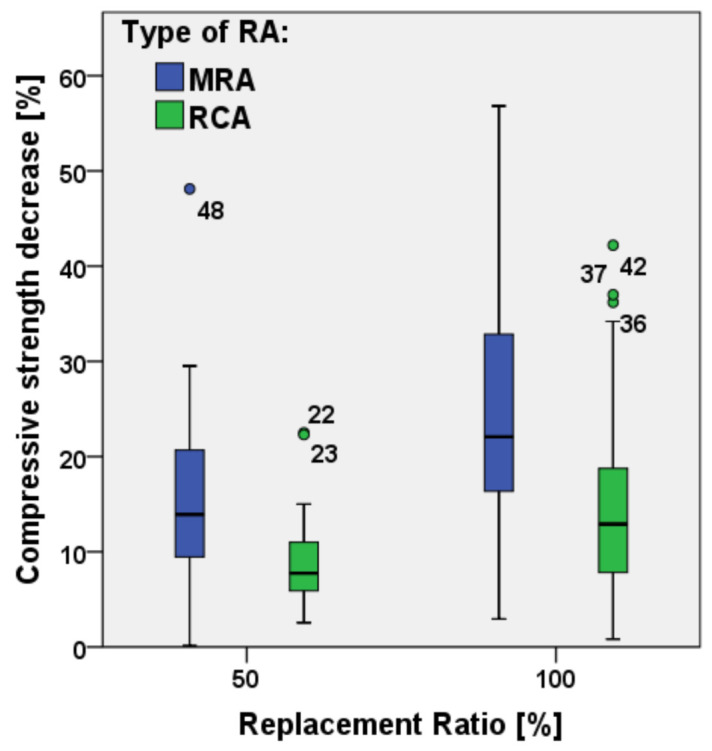
Comparison between MRAs and RCAs compressive strength decrease for replacement ratio of 50% and 100% [19,20,21,22,23,24,25,26,27,28,29,30,31,32,33,34,35,36,37,38,41,42,52,53,54,55,56,57,58,59,60,61,62,63,64,65,66,67,68,70,72].

By considering the difference between high and low-quality RAs, a possible explanation is the different distribution of particles coming from concrete with higher strength, which are less brittle and coarser at the end of the crushing process [31,64]. Another reason of the strength decrease is the increase of w/c ratio due to larger air gaps after hardening [28,29,45,56]. As far as the replacement percentage is concerned, Table 3 and Figure 11 show the maximum and minimum values of decrease of compressive strength for two different percentages of RA, namely 50% and 100%.

**Figure 12:**
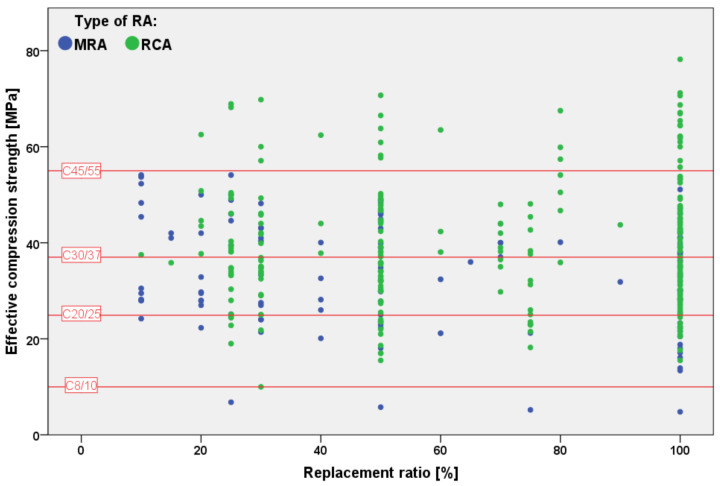
Effective final compressive strength of concrete made with MRAs and RCAs in relation with the replacement ratio [19,20,21,22,23,24,25,26,27,28,29,30,31,32,33,34,35,36,37,38,41,42,52,53,54,55,56,57,58,59,60,61,62,63,64,65,66,67,68,70,72].

It can be observed that, with 50% substitution, for MRAs the mean value of compressive strength decrease is about 15% while it is lower than 10% when RCAs are used. With 100% substitution of RA, the difference increases to about 22% and 13%, respectively. Figure 12 shows the effective compressive strength of the analyzed concretes after RA substitutions, as obtained from the available papers; once again, it can be observed a lower scatter of experimental results from concrete produced with RCAs only. It can also be noticed that concrete compressive strength can be higher than 45 MPa for most of the replacement ratios for concrete produced with RCAs while, to concrete produced with MRAs up to 25% of aggregate substitution, compressive strength can be higher than 35 MPa but it decreases for higher replacement ratios.

### 3.5. Other Mechanical Caractheristics

#### 3.5.1. Tensile Strength

A total number of 11 papers were considered for the analysis of tensile and flexural strength of concrete containing recycled aggregates [55]. Referring to the tensile strength, recycled aggregates with less attached mortar can be used as a substitute of natural aggregate, producing a concrete with a similar or even better performance [40]. In fact, it has been confirmed that a smoother surface of the recycled aggregates leads to better tensile properties [26]. The influence of adhered mortar on concrete properties has been discussed by some authors, who also found that concrete mixes containing aggregates with a more porous structure have a lower tensile strength [21,64].

**Figure 13:**
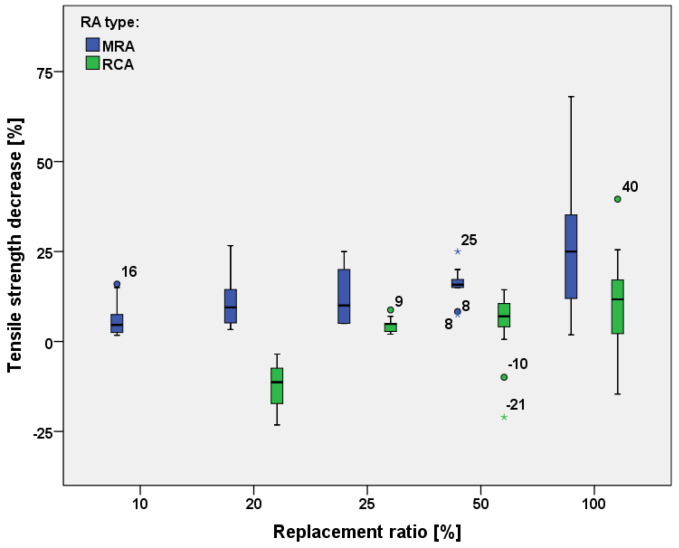
Comparison of the decrease of tensile strength between concrete containing recycled aggregates from mixed CDW and from recycle concrete only [19,21,22,24,35,40,41,55,60,65,66].

As expected, tensile strength also decreases for increasing values the w/c ratio [24,28,55], although this decrease is lower than that of the compressive strength [32]. Figure 13 shows the decrease of tensile strength in concrete made with recycled aggregates from mixed CDW and from concrete only. Table 4 defined the average decrease of tensile strength related to the replacement ratio.

Comparing recycled concrete with the natural one, the average decrease of tensile strength for concrete containing mixed recycled aggregate increases from 5% to 25% with increasing the recycled aggregate amount up to the total substitution. Some extreme values, represented by circles and asterisks, are present and represent values which rarely are found in the selected studies.

The average decrease of tensile strength for concrete containing MRAs increases from 5% to 25% when the amount of recycled aggregate increases up to total substitution. Conversely, a lower decrease of tensile strength is observed if aggregates from recycled concrete are used, showing a 5% decrease with total substitution.

#### 3.5.2. Elasticty Modulus

Similar results are observed for the elastic modulus since these mechanical properties are closely related to each other. Also in this case, by increasing the percentage of replacement of natural aggregate with recycled aggregate, the modulus of elasticity generally decreases [34,35,58]. This is mainly due to the increase in adhered mortar and microcracks on the surface on the recycled aggregates which leads to an increase in porosity [19,41,66]. Furthermore, Cordinalesi [70] found that elastic modulus decrease is higher for concrete produced with mixed recycled aggregates than recycled concrete aggregates and lower values were obtained with increasing the replacement ratio [72].

Also in this case, due to the characteristics of the RAs, some authors found that increasing w/c ratio from 0.4 to 0.5 leads to a decrease in elasticity modulus up to 12%, due to an excess of water after the hydration phase that can reduce the stiffness of the mortar phase in concrete. The latter does not occur if there was an increase of cement in the mixture [24,54].

### 3.6. Other Properties

Durability of concrete is the ability to maintain its integrity and its characteristics during the whole service life. As compared to concrete made with natural aggregates, due to the attached mortar, shrinkage, chloride penetration and freeze and thaw of RAs concrete can be reduced [77].

Referring to shrinkage, some studies demonstrate a correlation between the increase of shrinkage and the replacement percentage of natural aggregates with RAs [26,34,78], probably due to the mortar attached to the recycled aggregate [34,36]. Some authors observed the formation of shrinkage cracks during curing of concrete containing RAs [42]. Rashid et al. [31] found that shrinkage can be decreased using special additives in concrete with RAs, while Duan et al. [40] confirmed that concrete with high quality RA with low attached mortar had similar behavior as natural concrete.

**Figure 14:**
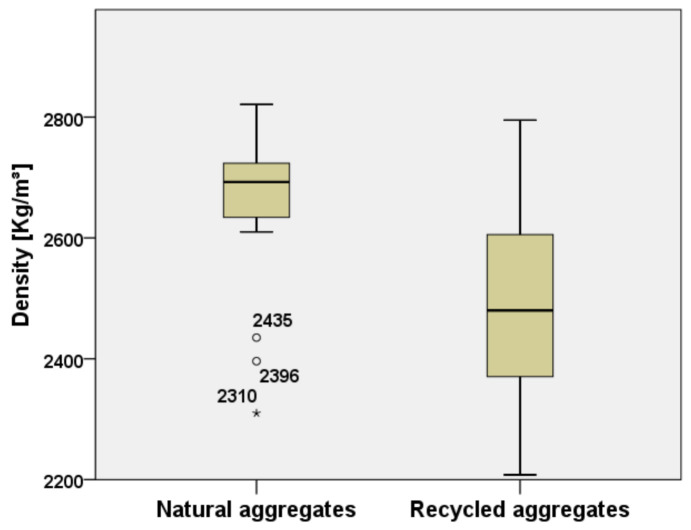
Comparison of the decrease of density between natural and recycled aggregates [20,21,26,29,32,33,34,35,36,37,38,41,42,66,67,72].

Density of concrete with RAs was also analyzed in 16 papers. Figure 14 shows results obtained from different studies and confirmed that density of RAs is lower than the density of natural aggregates and, consequently, concrete produced with RAs has lower density than the one with natural aggregates. In particular, as reported in the analyzed studies, the average density of RAs is about 2480 kg/m^3^ while the average density of the natural aggregates is about 2660 kg/m^3^ [19,20,26,30,31,43], the lower density is mainly due to the adhered mortar of RAs [56]. Moreover, some values, classified as outliers and extreme values, represented by circles and asterisks, can have lower values as 2310 kg/m^3^.

Lavano et al. [20] studied the influence of water/cement ratio on the density of concrete and found that it does not significantly affect the difference in density between the concrete with natural and recycled aggregates.

As far as chloride penetration is concerned, some authors observed that, by increasing the replacement ratio, the resistance of concrete to chloride penetration decreases due to the higher porosity of RAs [21,58,79]. Duan et al. [40] found that concrete with low mortar attached on RAs shows similar resistance to chloride penetration as natural concrete. Moreover, different studies proved that decreasing w/c can lead to a decrease chloride penetration [58,79].

Referring to the freeze and thaw resistance of concrete, contrasting results were found. Guo et al. [41] stated that freeze thaw performance for concrete with RAs is lower than conventional concrete, due to higher water absorption, porosity, and the presence of attached mortar. On the contrary, Yildirim et al. [79] found that concrete containing 50% of RAs is comparable to the natural one. Moreover, Tuyan et al. [80] stated that increasing w/c ratio could worsen the performance due to freezable water in concrete.

### 3.7. Leaching and Environmental Properties

Environmental behavior of concrete containing RAs was evaluated according to both leaching of pollutants from the recycled concrete and the whole sustainability of recycled concrete production. As concerns the leaching of pollutants, Diotti et al. [11,81] found that RCAs, due to the main presence of high quantities of cement, have an important release of total chromium with respect to the mixed one. On the other hand, MRAs have a high release of sulphates due to the presence of ceramic materials and gypsum. Galvin et al. [82] verified how the use of RA can lead to an increase in the release of pollutants from the monolithic concrete. The same authors, from the leaching test on RAs, observed a high level of release for all the regulated metals (Mo, Se, As, Sb, Cr, Zn, Cu, Ni, Pb, Cd, Ba). However, concrete blocks produced with different replacement percentages of natural aggregate with RAs (20%, 50% and 100%) showed a similar leaching behavior with respect to the control one.

Additionally, the analysis of the life cycle and the environmental impacts generated by the use or production of RA for concrete was investigated. Marinkovic et al. [68] found that the process of production of RAs has a slightly higher impact than natural aggregates. Conversely, Hossain et al. [83] found that the greenhouse gasses emission for the production of RAs is 58% lower than that of natural aggregate, while the phase of recycled concrete production drastically increased this impact due to the higher quantity of cement, required to reach the target mechanical properties. In fact, with 100% substitution with RAs the emissions of concrete were about 2% lower than the conventional one. Martinez-Lage et al. [70] determined the difference in environmental impact between the production of concrete with RCAs and MRAs and found an almost identical impact to that of natural concrete. The authors found that the impact increases with increasing the transportation distance of the RAs and the percentages of replacement.

## 4. Future Perspective

The paper presents a systematic review of literature published within the past 10 years (from 2010 to 2020) to provide an overview on the main characteristics of concrete with RAs. The results found in the literature survey presented in this paper show that a sustainable concrete requires specific rules.

First of all, the difference in properties of natural aggregates with respect to recycled aggregates from concrete only (RCA) and from mixed construction and demolition waste (MRA) is mainly driven by the presence of attached mortars on the surfaces of the aggregates. Mortar is responsible for the higher absorption, lower density and lower mechanical properties of RAs as compared to the natural one. The removal of attached mortar can increase the characteristics of recycled concrete quality, thus reducing the cement content for reaching the target strength; a possible solution is the increase of the crushing steps without creating further cracks on the surface. Due to the reduced strength of RA concrete, the compressive strength should be related to the environmental requirements; therefore, structural elements requiring lower strength can be made with RAs concrete while structural elements with higher strength should be made with concrete with natural aggregates.

From an environmental point of view, beside structural safety, designers should carry out LCA analyses in order to choose the best compressive strength to be adopted for structural elements. The maximum percentage of aggregate substitution should consider the availability of RAs in the surroundings of the construction site, in order to avoid inconvenient environmental costs. If RAs are not enough for a total replacement of natural aggregates, a lower replacement helps in increasing the concrete strength and reducing the cement content.

The use of pre-saturated RAs allows to increase concrete workability but this requires special attention to avoid changing the w/c ratio of the concrete with a consequent reduction of mechanical properties. A new generation of superplasticizers could be developed for concretes with RAs.

In any case, the quality of the initial CDW for the production of RAs is fundamental and it is possible through a selective demolition. It would allow to obtain higher quality aggregates that enhance the concrete quality which can be hardly reached with MRAs [84].

Leaching test on RAs lead to an increase of pollutants, but under the regulatory limits. In particular, the two types of RAs considered lead to a release of different pollutants, especially chloride from RCA and sulphates from MRAs. Leaching test from monolithic concrete blocks produced with RAs gives no significant results in terms of pollutants.

Referring to the economic impact from the use of RAs in construction, results show the difference between revenues and total costs that come into play throughout the life cycle of the product, concluding that downcycling is preferable to landfill. Furthermore, recycling after selective demolition presents a higher income even if demolition is more expensive. Finally, referring to the production of concrete, the most cost-efficient path is mobile recovery, in which cost savings are mainly achieved through a reduction in the transport and production of higher value-added and lower heterogeneity materials. In this context, results show that RAs are sustainable both from the environmental and economic point of view.

In a future perspective Xu et al. [85] demonstrate how by increasing the support of research studies and funds coming from national bodies, can anchorage the use of RAs in building construction.

## 5. Conclusions

The 42 selected papers evidence that, by increasing the substitution percentage of natural aggregates, concrete properties become worse. The studies, also demonstrate the importance of the quality of the type of RAs.

Regarding the water absorption of the two types of RAs analyzed (RCAs and MRAs), the experimental results highlight the importance of the selection during demolition for reducing water absorption. Experiments also demonstrate the importance of checking the attached mortar on the surface of the RAs; by increasing the number of crushing processes, the attached mortar reduces, thus enhancing the mechanical properties of concrete.

Referring to the water/cement ratio, results show the importance of RCAs, in order to enhance workability and limit the compressive strength decrease.

For concrete mechanical properties (in particular compressive strength), the importance of the quality of the RAs is observed and discussed since the reduction of compressive strength for concrete produced with RCAs is lower than that of concrete with MRAs. The attached mortar, the size and shape of MRAs are the main reason of the compressive strength decrease. Average compressive strength values of 35 MPa and 45 MPa are obtained using MRAs and RCAs respectively.

From the environmental point of view, the release of pollutants from concrete blocks is very low. However, concrete with RAs has a higher environmental impact evaluated by the life cycle assessment analysis, due to the higher cement dosage and RAs transportation 

## Figures and Tables

**Table 1:** Keyword summary.

Main Keyword	Secondary Keyword	Third Keyword
Concrete	Recycled Aggregates	w/c ratio
		Workability
	Recycled materials	Origin
		Dimension
	Recycled concrete	Compressive Strength
		Monolithic or tank test

**Table 2:** Average, maximum and minimum water absorption values.

	Natural Aggregate WA [%]	Recycled Aggregate WA [%]
Average value	1.71	5.13
Minimum	0.05	2.70
Maximum	5.20	14.70

**Table 3:** Maximum and minimum decrease of compressive strength for replacement ratio of 50% and 100% and type of aggregate.

Substitution Percentages and RA Type	Minimum Compressive Strength Decrease [%]	Maximum Compressive Strength Decrease [%]
50% MRAs	0.13	48.11
50% RCAs	2.54	22.50
100% MRAs	2.94	56.83
100% RCAs	0.81	42.20

**Table 4:** Average decrease of tensile strength related to replacement ratio and type of RA.

Type of RA	Minimum–Maximum Replacement Ratio	Tensile Strength Decrease	Reference
MRAs	10–100%	10–64%	[31]
MRAs	10–100%	2–13%	[54]
RCAs	25–100%	4–14%	[24]
MRAs	25–100%	5–35%	[60]
MRAs	10–100%	7–28%	[22]
RCAs	100%	6%	[65]
RCAs	100%	6%	[66]

## Data Availability

The data that support the findings of this study are available from the corresponding author upon reasonable request.
